# The Perceived Impact of the COVID-19 Pandemic on the Social Needs of Adult Emergency Department Patients

**DOI:** 10.1089/heq.2022.0015

**Published:** 2022-08-22

**Authors:** Timothy H. Gallagher, Kelly M. Doran, Elizabeth A. Samuels, Ryan P. McCormack

**Affiliations:** ^1^ Rush University Department of Emergency Medicine, Chicago, Illinois, USA.; ^2^ Ronald O. Perelman Department of Emergency Medicine and the Department of Population Health, NYU School of Medicine, New York, New York, USA.; ^3^ Department of Emergency Medicine, Alpert Medical School of Brown University, Providence, Rhode Island, USA.; ^4^ Ronald O. Perelman Department of Emergency Medicine, NYU School of Medicine, New York, New York, USA.

**Keywords:** COVID-19, emergency department, social needs

## Abstract

**Objectives:**

We aim to assess the influence of COVID-19 on the social needs of emergency department (ED) patients, and assess patients' access to social services.

**Methods:**

We conducted a cross-sectional survey of 175 purposively sampled adult ED patients.

**Results:**

Approximately half of participants stated that COVID-19 negatively impacted their social needs with statistically significant differences observed for race, ethnicity, and insurance status. Many participants did not know of available social services, and a majority welcomed assistance from the ED.

**Conclusion:**

This study suggests that unmet social needs have risen because of COVID-19, and EDs may be positioned to identify and assist affected patients.

## Introduction

Social determinants of health, the conditions in which people are born, grow, live, and work, shaped by the distribution of money, power, and resources, have a direct influence on the health of individuals and populations.^1^ Studies have repeatedly demonstrated that unmet social needs have an important effect on individuals' health and can produce health care inequities.^2^ Emergency departments (EDs) are an essential component of the health care safety net, and a primary site of medical care for people with unmet social needs.^3^

During the COVID-19 pandemic, communities have endured increased economic and personal strain resulting in increased unemployment, threatened and actual loss of housing, and increased food insecurity. Unemployment rates increased sharply, peaking at ∼15% in April 2020, from ∼4% pre-COVID-19.^4^ Owing to factors such as structural racism, types of employment, crowded living conditions, and limited health care access, individuals with high unmet social needs have been disproportionately impacted by COVID-19.^5^

The ED, as a critical health care entry point and social safety net for many people chronically or acutely impacted by unmet social needs, is uniquely positioned to identify changes in those needs, and may be a strategic venue to connect people with essential services. Few studies have yet to examine the social needs of ED patients during the COVID-19 pandemic, or how those needs were affected by the pandemic itself. In this study, we conducted survey questionnaires of ED patients to assess their social needs and the influence of the COVID-19 pandemic on those needs, as well as their access to social services and desire for ED-delivered assistance.

## Methods

We conducted a cross-sectional survey from June to August 2021 involving a purposive sample of adult ED patients presenting to two characteristically distinct large-volume New York City EDs—a private hospital in Brooklyn and a public hospital in Manhattan. Research staff verbally administered questionnaires to patients using REDcap, a secure web-based data management application; each questionnaire took ∼10 min. To help ensure the sample was representative of the overall ED population, we scheduled recruitment shifts on all days of the week, at various hours of the day (8 am to 10 pm), and evenly between sites during the 3-month period of medical student surveyors' availability.

Within those shifts, an online random number generator was used to sample active ED patients. In-person interpreters or hospital-approved telephone interpreters were used to communicate with patients whose primary language was not English. Patients were excluded if they were in medical extremis, prisoners, unable to communicate with the researcher or a hospital-approved interpreter, or unable or unwilling to verbally consent to participation.

The questionnaire included basic demographic information and questions about several domains of health-related social needs, including employment, housing, and food security, based on previously validated studies.^6,7^ No personal health information was collected. Participants were asked about their prior use of social services, knowledge about available services, their perception of how the COVID-19 pandemic impacted their social needs, and whether they considered the ED an appropriate site for addressing these needs (Supplementary Data).

All patients approached were offered a printed list of social service resources regardless of their participation in the study. Analyses were descriptive in nature. *Post hoc* testing of differences across the demographic groups was conducted using chi-squares for categorical variables using R software with bins of fewer than five collapsed. We report the raw *p*-values and state which variables are significant using the Benjamini–Hochberg procedure accepting a false discovery rate (Q) of 5%. The study was institutional review board approved.

## Results

In total, 175 questionnaires were completed: 53.7% at the public hospital and 46.3% at the private hospital. Table 1 summarizes baseline characteristics of all participants, the subset of participants who reported being negatively affected in one or more domains of health-related social needs as a result of the COVID-19 pandemic, and those who reported no change in health-related social needs.

**Table 1.** Demographics of All Participants, Participants Who Report Social Needs Affected by the COVID-19 Pandemic, and Participants Who Report Social Needs Unaffected by the COVID-19 Pandemic

	All surveyed patients, *N* (%), *n*=175	Surveyed patients with social needs affected by COVID-19, *N* (%), *n*=85	Surveyed patients with social needs not affected by COVID-19, *N* (%), *n*=90	*p*
Hospital				0.068
Public	94 (53.7%)	47 (55.3%)	47 (52.2%)	
Private	81 (46.3%)	38 (44.7%)	43 (47.8%)	
Age (years)				0.052
18–24	8 (4.6%)	3 (3.5%)	5 (5.6%)	
25–40	55 (31.4%)	30 (35.3%)	25 (27.8%)	
41–64	76 (43.4%)	41 (48.2%)	35 (38.9%)	
65+	36 (20.6%)	11 (12.9%)	25 (27.8%)	
Gender identification^a^				0.19
Male	85 (48.6%)	37 (43.5%)	48 (53.3%)	
Female	90 (51.4%)	48 (56.5%)	42 (46.7%)	
Race/ethnicity^b^				0.01^c^
American Indian or Alaska Native	1 (0.6%)	1 (1.2%)	0 (0.0%)	
Asian	14 (8.0%)	7 (8.2%)	7 (7.8%)	
Hispanic or Latinx	61 (34.9%)	37 (43.5%)	24 (26.7%)	
Middle Eastern or North African	5 (2.9%)	2 (2.4%)	3 (3.3%)	
Non-Hispanic Black or African American	37 (21.1%)	19 (22.4%)	18 (20.0%)	
Non-Hispanic White	43 (24.6%)	11 (12.9%)	32 (35.6%)	
Multiethnic	8 (4.6%)	5 (5.9%)	3 (3.3%)	
Other	2 (1.1%)	1 (1.2%)	1 (1.1%)	
Prefer not to answer	4 (2.3%)	2 (2.4%)	2 (2.2%)	
Highest level of education				0.042
Less than high school	38 (21.7%)	25 (29.4%)	13 (14.4%)	
High school or GED	67 (38.3%)	32 (37.6%)	35 (38.9%)	
College or associates degree	49 (28.0%)	23 (27.1%)	26 (28.9%)	
Education beyond college	18 (10.3%)	4 (4.7%)	14 (15.6%)	
Prefer not to answer	3 (1.7%)	1 (1.2%)	2 (2.2%)	
Insurance				<0.0001^c^
None	24 (13.7%)	16 (18.8%)	8 (8.9%)	
Public	102 (58.3%)	57 (67.1%)	45 (50.0%)	
Private	42 (24.0%)	8 (9.4%)	34 (37.8%)	
Combination public and private	4 (2.3%)	1 (1.2%)	3 (3.3%)	
Unsure/prefer not to answer	3 (1.7%)	3 (3.5%)	0 (0.0%)	

^a^ No respondents identified as nonbinary/prefer not to answer.

^b^ No respondents identified as Hispanic Black.

^c^ Met significance using the Benjamini–Hochberg procedure at the specified false discovery rate of 5%. Within race/ethnicity, *p*-value=0.01 for all race/ethnicity groups combined, 0.018 for Latinx/Non-Latinx, and 0.0005 for White/Non-White.

Across both sites, participants were 51.4% female, predominantly Latinx (34.9%), White (24.6%), or Black (21.1%), with a mean age of 49±16.8 years, which approximates the overall population demographics for each site. Thirty-five participants (20.0%) used an interpreter to communicate in Spanish; 10 participants (5.7%) used an interpreter for one of eight other languages. And 15.4% of participants reported a current or previous diagnosis of COVID-19.

Approximately half (85/175, 48.6%) of participants stated that the COVID-19 pandemic negatively impacted their employment, housing, and/or food security status. In *post hoc* analyses, race, ethnicity, and insurance status are associated with statistically significant differences in those reporting social needs being affected by the pandemic compared with those reporting being unaffected. Within those variables, affected participants were more likely to report being Latinx or non-White, and uninsured or not privately insured. Although education has a raw *p*-value of <0.05, it does not meet significance using the Benjamini–Hochberg procedure at the specified false discovery rate of 5% (Table 1).

Almost half of participants reported being either unemployed (42.9%) or underemployed (2.9%); 41.3% of whom stated the amount they worked for pay decreased because of the pandemic (Table 2). Less than a quarter (22.7%) of unemployed participants reported ever filing for unemployment.

**Table 2.** Selected Questionnaire Responses

	All surveyed patients, *N* (%), *n*=175	COVID-19 affected employment, *N* (%), *n*=36	Did not file for un employment, *N* (%), *n*=57	COVID-19 affected housing security, *N* (%), *n*=25	COVID-19 affected food security^a^, *N* (%), *n*=70	Reported past use of social services, *N* (%), *N*=52	Reported knowledge of available services, *N* (%), *N*=84	Reported ED appropriate setting for social services, *N* (%), *N*=149	Welcome social worker help in ED, *N* (%), *N*=119
Hospital									
Public	94 (53.7%)	20 (55.6%)	35 (61.4%)	17 (68.0%)	37 (52.9%)	31 (59.6%)	44 (52.4%)	80 (53.7%)	61 (51.3%)
Private	81 (46.3%)	16 (44.4%)	22 (38.6%)	8 (32.0%)	33 (47.1%)	21 (40.4%)	40 (47.6%)	69 (46.3%)	58 (48.7%)
Age (years)									
18–24	8 (4.6%)	1 (2.8%)	1 (1.8%)	0 (0.0%)	1 (1.4%)	2 (3.8%)	5 (6.0%)	8 (5.4%)	6 (5.0%)
25–40	55 (31.4%)	11 (30.6%)	15 (26.3%)	8 (32.0%)	27 (38.6%)	12 (23.1%)	27 (32.1%)	50 (33.6%)	41 (34.5%)
41–64	76 (43.4%)	21 (58.3%)	30 (52.6%)	12 (48.0%)	34 (48.6%)	26 (50.5)	37 (44.0%)	64 (43.0%)	50 (42.0%)
65+	36 (20.6%)	3 (8.3%)	11 (19.3%)	5 (20.0%)	8 (11.4%)	12 (23.1%)	15 (17.9%)	27 (18.1%)	22 (18.5%)
Gender identification^a^									
Male	85 (48.6%)	14 (38.9%)	30 (52.6%)	13 (52.0%)	31 (44.3%)	31 (59.6%)	40 (47.6%)	73 (49.0%)	64 (53.8%)
Female	90 (51.4%)	22 (61.1%)	27 (47.4%)	12 (48.0%)	39 (55.7%)	21 (40.4%)	44 (52.4%)	76 (51.0%)	55 (46.2%)
Race/ethnicity^b^									
American Indian or Alaska Native	1 (0.6%)	0 (0.0%)	0 (0.0%)	0 (0.0%)	1 (1.4%)	0 (0.0%)	0 (0.0%)	1 (0.7%)	1 (0.8%)
Asian	14 (8.0%)	3 (8.3%)	4 (7.0%)	1 (4.0%)	6 (8.6%)	1 (1.9%)	5 (6.0%)	11 (7.4%)	10 (8.4%)
Hispanic or Latinx	61 (34.9%)	19 (52.8%)	24 (42.1%)	7 (28.0%)	31 (44.3%)	18 (34.6%)	30 (35.7%)	54 (36.2%)	43 (36.1%)
Middle Eastern or North African	5 (2.9%)	0 (0.0%)	1 (1.8%)	0 (0.0%)	2 (2.9%)	0 (0.0%)	0 (0.0%)	5 (3.4%)	5 (4.2%)
Non-Hispanic Black or African American	37 (21.1%)	7 (19.4%)	17 (29.8%)	9 (36.0%)	16 (22.9%)	18 (34.6%)	22 (26.2%)	36 (24.2%)	23 (19.3%)
Non-Hispanic White	43 (24.6%)	4 (11.1%)	10 (17.5%)	5 (20.0%)	8 (11.4%)	12 (23.1%)	17 (20.2%)	29 (19.5%)	26 (21.8%)
Multiethnic	8 (4.6%)	2 (5.6%)	0 (0.0%)	2 (8.0%)	4 (5.7%)	3 (5.8%)	6 (7.1%)	8 (5.4%)	6 (5.0%)
Other	2 (1.1%)	0 (0.0%)	1 (1.8%)	1 (4.0%)	1 (1.4%)	0 (0.0%)	1 (1.2%)	2 (1.3%)	2 (1.7%)
Prefer not to answer	4 (2.3%)	1 (2.8%)	0 (0.0%)	0 (0.0%)	1 (1.4%)	0 (0.0%)	3 (3.6%)	3 (2.0%)	3 (2.5%)
Highest level of education									
Less than high school	38 (21.7%)	12 (33.3%)	18 (31.6%)	9 (36.0%)	23 (32.9%)	12 (23.1%)	16 (19.0%)	31 (20.8%)	21 (17.6%)
High school or GED	67 (38.3%)	10 (27.8%)	24 (42.1%)	7 (28.0%)	28 (40.0%)	26 (50.0%)	40 (47.6%)	61 (40.9%)	50 (42.0%)
College or associates degree	49 (28.0%)	10 (27.8%)	13 (22.8%)	6 (24.0%)	17 (24.3%)	9 (17.3%)	19 (22.6%)	42 (28.2%)	37 (31.1%)
Education beyond college	18 (10.3%)	3 (8.3%)	2 (3.5%)	2 (8.0%)	2 (2.9%)	4 (7.7%)	7 (8.3%)	14 (9.4%)	11 (9.2%)
Prefer not to answer	3 (1.7%)	1 (2.8%)	0 (0.0%)	1 (4.0%)	0 (0.0%)	1 (1.9%)	2 (2.4%)	1 (0.7%)	0 (0.0%)
Insurance									
None	24 (13.7%)	9 (25.0%)	15 (26.3%)	6 (24.0%)	15 (21.4%)	3 (5.8%)	11 (13.1%)	17 (11.4%)	10 (8.4%)
Public	102 (58.3%)	25 (69.4%)	36 (63.2%)	17 (68.0%)	46 (65.7%)	42 (80.8%)	53 (63.1%)	88 (59.1%)	68 (57.1%)
Private	42 (24.0%)	2 (5.6%)	3 (5.3%)	2 (8.0%)	5 (7.1%)	6 (11.5%)	17 (20.2%)	38 (25.5%)	36 (30.3%)
Combination public and private	4 (2.3%)	0 (0.0%)	2 (3.5%)	0 (0.0%)	1 (1.4%)	1 (1.9%)	3 (3.6%)	3 (2.0%)	3 (2.5%)
Unsure/prefer not to answer	3 (1.7%)	0 (0.0%)	1 (1.8%)	0 (0.0%)	3 (4.3%)	0 (0.0%)	0 (0.0%)	3 (2.0%)	2 (1.7%)

^a^ No respondents identified as nonbinary/prefer not to answer.

^b^ No respondents identified as Hispanic Black.

ED, emergency department.

Most survey participants had stable housing; however, 18.3% did not have a steady place to live and 8.6% worried about losing their housing. Among those reporting actual or fear of housing insecurity, 53.2% stated their housing became less stable because of the pandemic.

More than a third of participants (38.9%) worried that their food would run out before they got money to buy more, among whom 72.7% thought that their ability to buy food became harder because of the COVID-19 pandemic.

A majority of participants (69.0%) had not previously received help from a social worker or a social service organization and about half (46.0%) did not know about social service resources available to them. Among participants with one or more unmet social needs, 63.5% had never accessed social services in the past, and 49.4% were unaware of potential resources. Most participants (85.6%) reported that they thought it was appropriate for a health care provider in the ED to provide information about social services. This number rose to 90.6% among people with unmet social need(s). And 68.4% of all participants indicated they would like to speak with a social worker in the ED about services for health-related social needs. Again, this percentage was higher among participants with one or more unmet social needs (75.3%).

## Discussion

In this study, we found that nearly half of participants presenting to two contextually distinct NYC EDs had social needs negatively impacted by the COVID-19 pandemic. As consistent with findings from prior studies, we found that a large majority of participating patients in the ED indicated that it was appropriate for health care providers to address social needs.^8^ In contrast, a recent study utilizing a survey mailed to patients' homes showed that while most participants believed health systems should assist in addressing social needs, fewer people expressed a personal desire for such assistance.^9^

This discrepancy suggests the ED is a potential setting in which people are comfortable with both social needs screening and potential assistance. Importantly, data from this study show most participants with unmet social needs reported neither having accessed social services, nor knowing how to access them. Similarly, a large proportion of unemployed participants had not filed for unemployment benefits. A majority of the study participants were interested in speaking with an ED social worker. The finding that patients are not accessing such services and benefits, despite their interest in assistance, highlights the opportunity and importance of intervening in the ED, where they access care.

The outsized impact of the COVID-19 pandemic on communities of color has been tracked by the CDC.^10^ Although *a priori* formal testing of subgroups was not planned, *post hoc* analysis suggests disparities in social needs associated with race, ethnicity, and insurance status. ED interventions addressing social needs should prioritize these disproportionately affected populations.

This study also provides preliminary data to support the potential distribution of COVID-19-relief funds to EDs to identify and assist people negatively affected by the pandemic.

Limitations of this study include that it was conducted at EDs within a single metropolitan area; thus, its results may not be generalizable to all EDs nationwide. However, the disparate characteristics of the study sites as well as the considerable racial, ethnic, educational, and health care payer diversity of participants are strengths that partially mitigate this concern. Furthermore, the purposive sampling strategy helped ensure the sample was representative of the larger ED populations, though the lack of recruitment in overnight hours and outside the three summer months does pose some risk of missing population subsets.

The use of randomization to determine which patients were approached mitigates the risk of sampling bias. It is possible that use of an interpreter may have dissuaded some people from responding, however, 25.7% of respondents completed the questionnaire with the use of an interpreter. As with any staff-administered survey, responses may have been influenced by social desirability bias. An additional limitation in the interpretation of this study and its feasibility is that participation rates were not assiduously recorded by all staff who administered questionnaires. We estimate a 70% participation rate derived from a combination of documented rates when available and from staff recollection when not available.

This study did not establish whether the participants who indicated their social needs were negatively affected by the COVID-19 pandemic were referring to previously unexperienced needs, or to worsening of pre-existing needs. Lastly, as the study was primarily descriptive in nature without pre-specified hypotheses testing, the findings should be interpreted as exploratory. These results provide important preliminary data that warrant further study, which should include other ED contexts to broaden generalizability.

## Conclusion

As the COVID-19 pandemic continues to unfold, it will be important for EDs to confront the changing needs of their patient population. This study suggests that the burden of unmet social needs of many ED patients has risen substantially because of the COVID-19 pandemic, representing an opportunity for EDs to identify such patients, to offer assistance while they are in the ED, and to guide them to appropriate social services.

## Supplementary Material

Supplemental data

## Abbreviation Used

ED: emergency department
