# Supplementary material for: The Perceived Impact of the COVID-19 Pandemic on the Social Needs of Adult Emergency Department Patients
Source: Health Equity. 2022 Aug 22;6(1):610–5. doi: 10.1089/heq.2022.0015 (PMC9518808; doi:10.1089/heq.2022.0015)
Supplement: Supplemental data [file Supp_DataS1.docx]

**The Perceived Impact of the COVID-19 Pandemic on the Social Needs of Adult Emergency Department Patients**

**Questionnaire**:

Do you have a job?

- Yes (employed)
- Yes, but I don’t work as much as I would like to (under-employed)
- Yes, but I worry I may lose my job in the next 6 months
- No (unemployed)
- Other (ex: student, unpaid household work, retired, etc)
- Prefer not to answer

**Linked question (If unemployed or under-employed): Did the amount you work for pay decrease because of the COVID-19 pandemic?

- Yes
- Partly
- No
- I don’t know or prefer not to answer

**Linked question (If unemployed): Did you file for unemployment?

- Yes
- No
- I don’t know or prefer not to answer

**Linked question (If worried about employment): Are you worried you may lose your job because of the COVID-19 pandemic?

- Yes
- Partly
- No
- I don’t know or prefer not to answer

Do you have health insurance?

- Yes, public insurance such as Medicare or Medicaid
- Yes, private insurance
- Yes, a combination of public and private insurances
- No
- I don’t know or prefer not to answer

What is your living situation today?

- I have a steady place to live
- I have a place to live today, but I am worried about losing it in the future
- I do not have a steady place to live. (For example: I am staying with others, in a hotel, in a shelter, in a bus or train station, or outside)
- I don’t know or prefer not to answer

**Linked question: If Worried or Do Not have a steady place: Did your housing become less stable because of the COVID-19 pandemic?

- Yes
- Partly
- No
- I don’t know or prefer not to answer

Within the past year, were you ever worried that your food would run out before you got money to buy more?

- Yes, I worried
- No, I did not worry
- No, but I’m worried that in the next few months my food may run out before I get money to buy more
- I don’t know or prefer not to answer

**Linked question: If worried, or Yes: Did your ability to buy enough food become harder because of the COVID-19 pandemic?

- Yes
- Partly
- No
- I don’t know or prefer not to answer

Within the past year, the food you bought just didn’t last and you didn’t have the money to get more.

- Often true
- Sometimes true
- Never true
- I don’t know or prefer not to answer

**Linked question: If often or sometimes true: Did your ability to buy enough food become harder because of the COVID-19 pandemic?

- Yes
- Partly
- No
- I don’t know or prefer not to answer

In the past, have you ever received help from a social worker or other social service organization with jobs, housing, or food?

- Yes (please list organization(s) if able)
- No
- I don’t know or prefer not to answer

Do you think COVID has affected your life already or may affect your life in the future and cause you to seek help with finding jobs, housing, or food?

- Yes
- No
- Maybe
- I don’t know or prefer not to answer

If you do want help with jobs, housing, or food now or in the future, do you know of any social workers, social services organizations, or resources available to you?

- Yes (please list if able)
- No
- Not applicable

Do you think it is appropriate for a health care provider in the emergency department to give you information on social services they think may be helpful to you?

- Yes
- No
- Maybe

Now, or in the future, would you like a social worker to talk with you while you are in the emergency department about social services that your doctor thinks may be helpful to you?

- Yes
- No
- Maybe
